# First Determination of Glycidyl Ester Species in Edible Oils by Reverse-Phase Ultra-Performance Liquid Chromatography Coupled with an Evaporative Light-Scattering Detector

**DOI:** 10.3390/molecules26092702

**Published:** 2021-05-05

**Authors:** Ping-Yi Wu, Hsuan Chen, Nan-Wei Su, Tai-Ying Chiou, Wei-Ju Lee

**Affiliations:** 1 Master Program in Food Safety, Taipei Medical University, Taipei 11031, Taiwan; ma47106006@tmu.edu.tw (P.-Y.W.); ma47107003@tmu.edu.tw (H.C.); 2 Department of Agricultural Chemistry, National Taiwan University, Taipei 10617, Taiwan; snw@ntu.edu.tw; 3 School of Regional Innovation and Social Design Engineering, Kitami Institute of Technology, Koen-cho, Kitami, Hokkaido 090-8507, Japan; tkyuu@mail.kitami-it.ac.jp; 4 School of Food Safety, Taipei Medical University, Taipei 11031, Taiwan; 5 Research Center of Food Safety Inspection and Function Development, College of Nutrition, Taipei Medical University, Taipei 11031, Taiwan

**Keywords:** glycidyl ester species, edible oil, C18 column, validation, UPLC-ELSD

## Abstract

In this work, a new ultra-performance liquid chromatograph-evaporative light-scattering detector (UPLC-ELSD) method for quantitation of glycidyl esters (GE) contents in edible oils is presented. The method features complete separation of five GE species within 20 min by a C18 column and gradient elution with a mobile phase consisting of 85% and 2.5% methanol aqueous solutions. The coefficients of regression (R^2^) were all ≥0.9999 for the linear-quadratic regression curves of GE species in a concentration range of 5~80 μg/mL. The intraday and interday recoveries (%) of GE species in solvent were in a range of 81.3~107.3%, and the intraday and interday coefficients of variation (CVs, %) were all ≤8.6%. The average recovery (%) of GE species spiked in extra-virgin olive oil samples ranged from 88.3~107.8% and the intermediate precision (CV, %) of ≤14% indicated acceptable accuracy and precision. The method exhibited limit of quantification (LOQ) for each GE species (0.6 μg glycidol equivalents/g oil). The method was applied to determine GE concentrations of six commercial oil samples, and total glycidol equivalents were consistent with data obtained by GC-MS method. This UPLC-ELSD method could be adopted for precursory screening and research purposes to improve food safety when MS detectors are unavailable.

## 1. Introduction

Glycidyl esters (GEs) are a class of food-processing contaminant widely found in edible oils and fats and oil-based food products [1,2,3,4]. Oil materials are extracted by organic solvents to obtain crude oils which are subjected to refining processes in order to improve their quality and stability by removing impurities, such as phospholipids, free fatty acids, color pigments, and odorants. During the deodorization step of the refining process, GEs are generated under high temperatures (210~260 °C) [5,6,7,8,9]. Diacylglycerols (DAGs) and monoacylglycerols (MAGs) are known to be precursors of GEs via free radical-mediated mechanisms involving a cyclic acyloxonium intermediate [4]. Thus, chemical structures of GEs contain a common terminal epoxide group and bear different fatty acid compositions, which may remain from the acylglycerols in edible oils. In addition, GEs are abundantly found in refined oils, especially in palm oils, because of their high deodorization temperature and precursor amounts [4,6,7,9,10].

Although there is insufficient evidence showing that GEs have negative effects on human health, recent toxicological assessments revealed that glycidol, the hydrolysate liberated from GEs, is of great safety concern. Glycidol is recognized as being neurotoxic, mutagenic, and toxic for reproduction according to in vitro and in vivo animal studies [11,12]. Glycidol is also a genotoxic carcinogen, since its epoxide structure reacts with DNA to form DNA adducts, and is classified in group 2A (probably carcinogenic to humans) by the International Agency for Research on Cancer (IARC) [11]. Accordingly, risk assessments of GEs were conducted based on the margin of exposure (MoE), and consumers’ intake should be “as low as reasonably achievable” (ALARA) [13]. The European Commission has set maximum limits for GEs in vegetable oils and fats placed on the consumer market and as ingredients in food when used for the production of baby food and processed cereal-based food for infants and young children as 1 and 0.5 μg glycidol equivalents/g oil, respectively [13].

Still no specific method for determining GEs in oils and fats is prescribed, and laboratories are recommended to select fully validated methods of analysis for the respective matrices. Over the past decade, multiple analytical methods, which can be divided into indirect methods and direct methods, were reported for the quantitation of GEs in oils and fats [4]. Based on structural similarities of GEs, the principle of indirect analysis is to hydrolyze intact GEs into glycidol under acidic, alkaline, or enzymatic treatment, followed by purification, derivatization, and quantitation by gas chromatography-mass spectrometry (GC-MS) [4,14]. The total amount of GEs determined by indirect methods is therefore expressed as a glycidol equivalent concentration. Currently, official American Oil Chemists Society (AOCS) methods include Cd 29a-13 (unilever method), Cd 29b-13 (3-in-1 method), Cd 29c-13 (difference method), and Cd 29d-13 (enzymatic method) [15]. The EU Commission recommends using AOCS official methods for analysis of GEs and 3-monochloro-propanediol esters (3-MCPDEs) in edible oils and oil-containing foods. Among them, the AOCS Cd 29c-13 method has the shortest analysis time, while Cd 29a-13 method is time-consuming but obtains the most accurate results [15].

Direct methods measure the level of each GE species in edible oils which have only undergone a sample purification procedure prior to liquid chromatography (LC)-MS [16,17,18] or LC-tandem-MS (LC-MS/MS) analysis [10,19]. Double solid-phase extraction (SPE) with the use of octadecyl bonded and unbonded silica-based sorbents is employed to remove impurities and the matrix in edible oil [10,16,17,18,19]. Moreover, Haines et al. exploited LC-time of flight-MS (LC-TOF-MS) to determine GEs without sample purification pretreatment [20]. Direct methods seem to be an ideal way for GE quantification because they are exempt from issues of incomplete transesterification and interconversion between GEs and 3-MCPDEs found in some indirect methods [4,21]. Nevertheless, an extensive range of GE standards and internal standards are required for indirect analyses, which are costly and some of them are commercially unavailable [4,14].

As a universal detector, evaporative laser-scattering detectors (ELSDs) have been widely used in lipid analysis, because poor ultraviolet (UV) absorbance, solvent selection, and gradient elution do not restrict its application [22]. In our previous studies, triacylglycerol (TAG) species and phosphatidylcholine (PC) species were successfully analyzed by reverse-phase HPLC-ELSD [23,24]. Compared to MS detectors, due to the absence of matrix effects, internal standards are not necessary when using ELSD. In contrast, ELSD is a non-specific detector with lower sensitivity, so it is crucial to completely dissolve the peaks to obtain accurate determinations and prevent interference. As a result, identification of peaks can be achieved by specific retention times of respective GE species. In our previous studies, TAG and PC species esterified with various fatty acids were satisfactorily separated by a C30 column and a binary solvent system [23,24]. Considering the similar structures of GEs which differ in only one fatty acyl chain, ultra-performance liquid chromatography (UPLC) is the detector of choice due to the significant advantages in resolution, speed, and sensitivity over HPLC.

Hence, the aim of this study was to apply UPLC-ELSD as a simple and cost-effective tool to quantitate five main GE species in edible oils. Double SPE cartridges were used to clean-up and concentrate GEs prior to the UPLC-ELSD analysis. Chromatographic conditions were adapted and modified to achieve separation of five GE species. The developed method was validated according to Taiwan Food and Drug Administration (TFDA) specifications to demonstrate the linearity, sensitivity, accuracy, and precision. The feasibility was then evaluated by determining actual samples and comparing the results obtained with the official AOCS method, Cd 29a-13.

## 2. Results and Discussion

### 2.1. Development of the UPLC-ELSD Method

**Figure 1:**
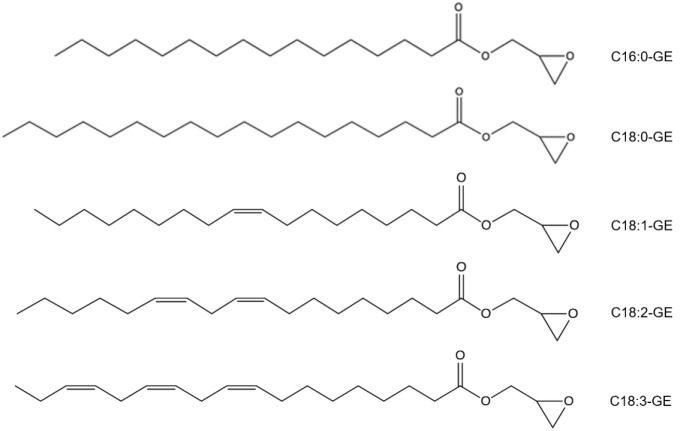
Chemical structures of five glycidyl ester (GE) species.

**Figure 2:**
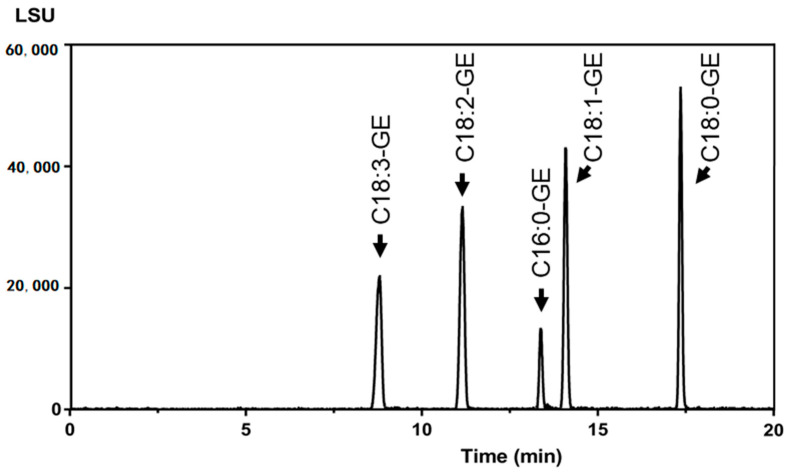
The UPLC-ELSD chromatogram of GE standard mix including C16:0-GE, C18:0-GE, C18:1-GE, C18:2-GE, and C18:3-GE.

During the development process, the challenge was to properly separate five GEs that share a similar chemical structure (Figure 1) in order to attain adequate discrimination of their responses on non-specific ELSD chromatograms to allow precise quantification. Four modified LC conditions were tested combining C18 columns as the stationary phase and different mobile phases as presented in Table 1. As shown in Table 2, with the conditions employed, the eluting sequences for GEs were as follows: C18:3-GE, C18:2-GE, C16:0-GE, C18:1-GE, and C18:0-GE, indicating that the retention times of GE species were affected by their chemical structures. The influencing factors included the length of the carbon chain and the number of double bonds. GEs with either a longer carbon chain or fewer double bonds had stronger intermolecular interactions with octadecyl carbon chains packed on the C18 column, similar to TAG and PC species [23,24]. In Table 2, C18: 3-GE with three double bonds was eluted first while C18: 0-GE exhibited the longest retention time. In addition, the BEH C18 column had better resolution of the five GE compounds compared to the CSH C18 column. The major obstacle of all tested conditions was to separate C16:0-GE and C18:1-GE, since they were eluted with very similar retention times (Table 2). The separation could only be achieved in condition no. 4 through a high-polarity solvent system composed of MeOH:water 85:15 (*v*/*v*) (solvent A) and MeOH:water 2.5:97.5 (*v*/*v*) (solvent B) to prolong retention times and a C18 BEH column of 15-cm length for extra resolution (Table 2). In addition, for better sensitivity, parameters of the nebulizer gas pressure and drift tube temperature were respectively set at 50 psi and 55 °C, because the intensity of the signal was enhanced with a higher flow rate or lower temperature [25]. The resultant chromatogram of the five GE species is shown in Figure 2. No peak was found in the chromatogram of the blank, whereas five peaks of individual GE species were completely resolved within 8.3~16.6 min.

### 2.2. Validation of the UPLC-ELSD Method

The calibration curves of GE species were achieved by plotting concentrations of each analyte against their corresponding mean peak areas. The equations of each regression curve and coefficients of determination (R^2^) are shown in Table 3. A quadratic regression model was found to be more applicable than a linear regression model for GE species by showing better coefficients of determination, consistent with the known fact that the ELSD response was not linear [23,26]. The coefficients of determination were >0.9999 for all GE species, attesting that the method was reliable for GE quantitation (Table 3). In accordance with guidelines for the validation method issued by the TFDA, R^2^ > 0.99 is considered evidence of acceptable fit of the data to the regression line [27].

The LOD and LOQ values were determined by injecting a series of diluted stock solutions containing very low concentrations of GEs to obtain S/N ratio values of 3 and 10, respectively. The S/N was directly determined by the chromatographic software. The highest LOD and LOQ values of 2.4 μg/mL and 8.0 μg/mL determined for C16:0-GE were respectively set as the instrumental LOD and LOQ (Table 3). As can be seen in Figure 2, a smaller peak was detected for the lowest molecular weight compound (i.e., C16:0-GE) even when all were prepared at the same concentration, because it was more likely to be nebulized and evaporated. Therefore, the peak of C16:0-GE had the lowest intensity among all the GE species, about 2-times lower than other GE species. Using C16:0-GE as a benchmark, the LOQ of this method for each GE was all set as 2.5 μg/g oil due to a 3.2-fold concentration increase in the sample preparation process.

The intra-day precision gives the distribution of duplicate measurements for three concentration levels of each GE species, providing a total of six determinations. For inter-day precision, analyses were further performed for another 2 days and 12 analyses in total were obtained. In this work, CV (%) was used as a measure of precision. The presented method had a precision of ≤2% for intra-day precision and ≤9% for inter-day precision (Table 4), showing a good precision for each GE analyzed. The accuracy of the method was determined by spiking the known amounts of standards at three identical concentrations. Intra-day recovery values were in the range of 97.2~107.3%, and inter-day recovery values ranged from 81.3~107.1%, demonstrating good method performance (Table 4).

A recovery study was further conducted by spiking the oil matrix with known amounts to GE standards. Unrefined extra-virgin olive oil is commonly used as the oil matrix for GE analytical method development due to its rare endogenous GEs, which are mainly formed under high-temperature heat treatment during the deodorization step of oil refining [5,6,7,8]. Although unrefined olive oil contains other components with similar polarities to GEs, no interfering substances were found in the chromatogram at the retention times of any GE species. Five distinct peaks were separated in the chromatogram of extra-virgin olive oil sample. According to TFDA specifications for validation of an analytical method, for samples with an analyte concentration range of 1~10 ppm, recovery should fall within the range of 75~120% [27]. As shown in Table 5, the mean intermediate accuracy ranged from 88.3~107.8%, demonstrating good selectivity and suitability of the analytical method for determining GE species in this oil matrix. Moreover, the intermediate precision results (CV, %) also met the requirements of TFDA specifications that all values be <14% (Table 5) [27]. Repeatability was also evaluated by retention time variation of the five GE species under optimized condition. The coefficients of variation (CVs, %) achieved for all analytes were ≤2.6% (Table 5).

### 2.3. Sample Analysis by the UPLC-ELSD Method and GC-MS Method

**Figure 3:**
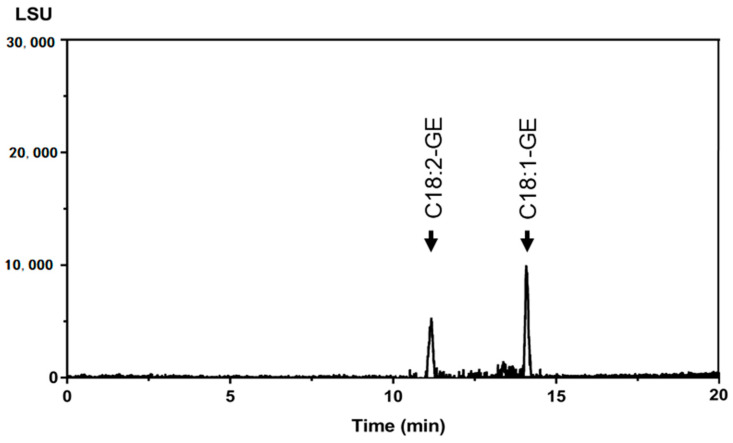
The UPLC-ELSD chromatogram of a rice bran oil sample.

In this study, the validated UPLC-ELSD method was applied to determine GE contents in six commercial refined oils. The UPLC-ELSD chromatogram of the rice bran oil sample is shown in Figure 3 as an example. As listed in Table 6, the GE levels of two palm oils and one rice bran oil were quantified by the UPLC-ELSD method, whereas the rest were determined to be below the LOQ for all GE species. The glycidol equivalents of oil samples were further calculated from GE levels by dividing the corresponding molecular weight and summing them up; they ranged from 1.41 ± 0.08 to 5.03 ± 0.53 µg/g. Additionally, the AOCS Cd 29a-13 method was employed to determine reference values of GE contents for the six oil samples. As can be seen in Table 6, values obtained by this method did not significantly deviate from the actual concentrations determined by GC-MS. Relative percentage differences between the concentrations of determined values and reference values were ≤15%. It complied with the criterion that all values were within 30% of reference values except for cases which were below the LOQ, indicating this validated method is reliable for GE quantitation of refined edible oils [27]. It was noted that some minor components were present in unrefined oil matrices that may have interfered with the measurement. However, the recovery and sample measurement results showed that the influence of the oil matrix was negligible owing to the high resolution of UPLC separation. Additionally, impurities in the refined oils were less likely to have interfered with the GE peaks (Figure 3).

In Table 6, the relative percent differences between two methods could mainly be attributed to the latent amount of other GE species which were determined to be below the LOQ by this direct UPLC-ELSD method, while all GE species were measured by the indirect GC-MS method. The GE contents of palm oil were mainly composed of two GE species, namely C16:0-GE and C18:1-GE, which was paralleled to its fatty acid composition of palm oil, with oleic acid (C18:1) as the dominant fatty acid, followed by palmitic acid (C16:0) [27]. These results were consistent with previous studies which showed that the main fatty acyl groups of GE species in palm oils are similar to fatty acid compositions of TAGs, since GEs are formed from the acylglycerol products of TAGs by a hydrolysis-pyrolysis process [2,17,20]. The same trend was also found for the rice bran oil sample, where only the major C18:1-GE and C18:2-GE species were measurable [28]. The glycidol equivalents should be converted from each GE concentration based on its molecular weight ratio. When the GE species is determined to be under LOQ by this method, it means the GE species was less than 2.5 μg/g oil. Considering the molecular weight ratios of GE species to glycidol range from 4.23 to 4.61, the method LOQ for individual GE species was ~0.6 μg glycidol equivalent/g oil. However, the stringent requirement on LOQ set by European Food Safety Authority (EFSA) for GE analytical methods had to be at 0.1 mg/kg or lower (expressed as glycidol), impairing the suitability of this method for commercial usage. The presence of trace GE species in oil samples could be confirmed by the aid of further LC-MS studies with no need for sample pretreatment.

The varied GE levels in different edible oils depend on several factors, such as the quality of the oil material, precursor amounts, and the deodorization duration and temperature. Generally, palm oil has the highest contents of GEs among all edible oils, followed by rice bran oil [4,10,20]. GEs are higher in the oils and fats produced by oil fruits (e.g., palm and olive) and rice bran than oil seeds, since these oil materials are more vulnerable to hydrolysis under storage conditions, resulting in higher contents of GE precursors (i.e., DAGs and MAGs) [4,8]. Moreover, considering the concomitant high free fatty acid levels in these crude oils, a physical refining process that features higher temperatures in the deodorization process than chemical refining to remove free fatty acids is normally used for palm oil and rice bran oil. GE contents of palm oil and rice bran oil could be consequently quantified in UPLC-ELSD but not the other oils (Table 6). In addition, these are also the oil samples that exceed statutory GE limit regulated by EU that the GE contents of vegetable oils and fats placed on the market for the final consumer should be ≤1 μg glycidol/g of oil [13]. Other edible oils (i.e., sunflower oil, safflower oil, and canola oil) contained glycidol equivalent less than 1.0 µg/g oil (Table 6), which was similar to values reported in the literature [4,10]. Additionally, since glycidol equivalent of the extra-virgin olive oil sample was determined to be <LOQ, where the LOQ value of the GC-MS method was 0.05 µg/g oil, it was suitable to be used as a blank oil matrix in validation experiments.

To control the levels of GEs in oils and fats, validated methods of analysis for GEs can be selected by laboratories on the basis of their pros and cons as well as varying scopes and applications. The limitations of this study were the characteristics of the methodological design which influenced the application and interpretation of the obtained results. In the present study, only five main GE species were considered; therefore, those oils and fats primarily composed of medium-chain fatty acids were not investigated. This alternative indirect method could be used for GE measurement in refined palm oils or other edible oils with relative high amount of GEs and research purposes to monitor the changes of main GE species simultaneously without the need of MS detectors.

## 3. Materials and Methods

### 3.1. Materials and Chemicals

Seven edible oil samples were randomly purchased from local supermarkets (Taipei, Taiwan). Reference standards including glycidol palmitate (C16:0-GE, purity 98%), glycidol stearate (C18:0-GE, purity 96%), glycidol oleate (C18:1-GE, purity 98%), glycidol linoleate (C18:2-GE, purity 98%), glycidol linolenate (C18:3-GE omega-3, purity 85%), glycidyl palmitate-d5 (C16:0-GE-d5, purity 97%), rac 1,2-bis-palmitoyl-3-chloropropanediol (C16:0-C16:0-3-MCPDE, purity 98%), and rac 1,2-bis-palmitoyl-3-chloropropanediol-d5 C16:0-C16:0-MCPDE-d5, purity 98%) were purchased from Toronto Research Chemicals (Toronto, Canada). Reagents were all HPLC grade purchased from either Merck (Darmstadt, Germany) or J.T. Baker (Phillipsburg, NJ, USA). Chemicals such as phenylboronic acid (PBA) were bought from Sigma-Aldrich (St. Louis, MO, USA). The Sep-Pak ac RC C18 cartridge 10 g and Sep-Pak ac RC silica cartridge 10 g were purchased from Waters (Milford, MA, USA).

### 3.2. UPLC Instrumentation and Chromatographic Conditions

All chromatographic analyses were performed with a Waters ACQUITY UPLC coupled with a Waters 2424 ELSD (Waters). The chromatographic conditions reported by MacMahon et al. (2013) and Hori et al. (2012) were modified and implemented in the UPLC system [10,16]. In all, four UPLC conditions were tested in this study as listed in Table 1. The ACQUITY UPC2 columns featuring Ethylene-Bridged Hybrid (BEH) (2.1 × 100 mm or 2.1 × 150 mm, 1.7 µm, 130Å) and Charged-Surface Hybrid (CSH) (2.1 × 100 mm, 1.7 µm, 130Å) were selected for their good selectivity and peak shape. The column temperature was set to 30 °C. The binary mobile phase used was composed of acetonitrile (ACN) and isopropyl alcohol (IPA), or different ratios of aqueous methanol solutions. The flow rate was 0.25 mL/min, and the injection volume was 10 µL. The nebulizer temperature and drift tube temperature of the ELSD detector were set to 30 and 55 °C, respectively, and the nebulizer gas pressure was set to 50 psi.

### 3.3. Oil Sample Pretreatment

A clean-up procedure based on a double SPE approach was employed for all oil samples. Briefly, 1 g of oil sample was accurately weighed and dissolved in 10 mL of tert-butyl methyl ether/ethyl acetate (4:1, *v*/*v*). A 4-mL aliquot of sample solution was transferred to a series of two Sep-Pak C18 Vac RC cartridges (10 g sorbent per cartridge) that had previously been conditioned with 40 mL MeOH. The sample solution was eluted with 3 × 40 mL MeOH, at the elution rate of 1 drop/s using vacuum, as needed. The eluate was collected in the flask and evaporated under vacuum. The obtained residue was dissolved in 20 mL of hexane/ethyl acetate (95:5, *v*/*v*) and loaded onto another series of two Sep-Pak silica Vac RC cartridges (10 g sorbent per cartridge) which had been pre-conditioned with 40 mL n-hexane/ethyl acetate (95:5, *v*/*v*). Again, the cartridges were eluted with 3 × 40 mL n-hexane/ethyl acetate (95:5, *v*/*v*) and the eluate was collected in a flask. The elution rate was maintained at 1 drop/s using vacuum, as needed, and the eluate was evaporated under vacuum. The dried residue was dissolved in 1 mL isopropanol and filtered with a 0.22-µm hydrophilic polyvinylidene difluoride (PVDF) membrane (CNW^®^ Technologies, Shanghai, China). The filtrate was dried with a N_2_ gas stream and dissolved in isopropanol at a final volume of 50 μL for the UPLC-ELSD analysis.

### 3.4. Method Validation

The developed UPLC-ELSD method was validated with respect to calibration, sensitivity, accuracy, and precision. Standard stock solutions were prepared with five reference compounds by dissolving 10 mg into 10 mL ACN, and further diluting this to various concentrations (5, 10, 20, 40, 50, and 80 μg/mL) in triplicate. Calibration curves were constructed by plotting the mean chromatographic peak area versus the concentration of the standard solution for each analyte. The sensitivity of this method was determined by injecting a series of appropriate concentrations of the diluted solutions. The instrumental limit of detection (LOD) and limit of quantification (LOQ) were respectively defined as 3- and 10-times the signal-to-noise ratio (S/N). Three concentrations (80, 50, and 40 μg/mL) were replicated on the same day (day 1) and another two on consecutive days (days 2 and 3) to determine the inter-day and intra-day precision and accuracy. The intermediate precision and accuracy were determined by analyzing five GE species in oil samples in quintuplicate by different analysts on different days. An aliquot of 1 g of blank olive oil was dissolved in 10 mL ACN spiked with GE standards at concentrations of 6.25 and 12.5 µg/mL, and subjected to the same sample preparation procedure mentioned above to give final concentrations of 20 and 40 µg/mL, respectively.

### 3.5. GE Determination by GC-MS

The determination of GEs was carried out as described in the Cd 29a-13 AOCS official method [15]. A portion of 0.1 g of the oil samples was weighed into a 10-mL glass tube with the addition of 100 μL mixed internal standard solution (containing C16:0-GE-d5 corresponding to 2.5 μg/mL of glycidol) and dissolved in 2 mL of tetrahydrofuran (THF). A volume of 30 μL of an NaBr acid aqueous solution (3.3 mg/mL, 5% H_2_SO_4_) was applied to convert the GEs to 3-monobromo-1,2-propanediol esters (3-MBPDEs). The mixture was homogenized and incubated at 50 °C for 15 min. The reaction was stopped by the addition of 3 mL 0.6% NaHCO_3_ (*w*/*v*), and the target compounds were extracted with 2 mL n-heptane. The upper layer was transferred to an empty glass tube and evaporated at 40 °C with a stream of N_2_ and the residue was dissolved in 1 mL of THF. The transesterification reaction was performed at 40 °C for 16 h after adding 1.8 mL 1.8% sulfuric acid solution in methanol (*v*/*v*) to the THF solution. The reaction was stopped by the addition of 0.5 mL 9% NaHCO_3_ (*w*/*v*), and the organic solvents were evaporated at 40 °C under a N_2_ stream. Fatty acid methyl esters were removed from the sample by adding 2 mL of 20% Na_2_SO_4_ (*w*/*v*), followed by liquid-liquid extraction with 2 × 2 mL of heptane. Free-form 3-MBPD was then derivatized with 200 μL of a 250 mg/mL phenylboronic acid (PBA) solution (acetone:H_2_O 19:1, *v*/*v*) incubated in an ultrasonic bath at room temperature for 5 min. PBA derivatives were extracted with heptane (2 × 1 mL), and evaporated at 40 °C with a stream of N_2_. The residue was re-dissolved in 400 μL n-heptane, and filtered through a 0.22-µm hydrophilic PVDF membrane.

GC-MS analyses were carried out on an Agilent 7820A GC equipped with an Agilent 5977b inert single quadrupole MS (Agilent Technologies, Santa Clara, CA, USA) operated in electron ionization (EI) mode at 70 eV. Separation was achieved using a DB-5MS capillary column (30 m × 250 μm × 0.25 μm, Agilent Technologies). An aliquot of 1 μL of the sample extract was injected into the split/split-less injector in a pulsed split-less mode at 250 °C. Helium was used as the carrier gas at a constant flow rate of 1.2 mL/min. The transfer line temperature was set to 300 °C. The oven temperature program was as follows: initial temperature of 80 °C held for 1 min, from 80 to 120 °C at 10 °C/min, held for 1 min, from 120 to 156 °C at 3 °C/min, and from 156 to 300 °C at 36 °C/min, held for 9 min. The target analytes were detected in the selected ion monitoring (SIM) mode. The following ions were monitored: m/z 147 and 196 for 3-MCPD, m/z 240 and 147 for 3-MBPD, m/z 150 and 201 for the 3-MCPD-d5 internal standard, and m/z 245 and 150 for the 3-MBPD-d5 internal standard. All samples were determined in triplicate. Results for GEs are expressed in equimolar amounts of glycidol (glycidol equivalents).

## 4. Conclusions

In this work, we successfully developed a new UPLC-ELSD method for quantitating individual GE species in refined edible oils. The selectivity of this method was achieved through complete separation of five target GE species using an optimized reverse-phase UPLC system equipped with a C18 column. The developed method was validated in terms of fitted quadratic regression curves, sensitivity, precision, and accuracy, and all results met the acceptance criteria of the official method validation guidance. The analytical performance was comparable to GC-MS method when determining total glycidol equivalents of actual oil samples, demonstrating this method was suitable to quantify individual GE species containing glycidol equivalents higher than 0.6 μg/g oil and could only be applied for precursory screening and research purposes.

## Figures and Tables

**Table 1:** UPLC-ELSD conditions for glycidyl ester (GE) determination.

Condition	1			2			3			4		
Stationary phase	CSH C18 column 2.1 × 100 mm, 1.7 µm			BEH C18 column 2.1 × 100 mm, 1.7 µm			BEH C18 column 2.1 × 150 mm, 1.7 μm			BEH C18 column 2.1 × 150 mm, 1.7 μm		
Mobile phase	Time (min)	A: ACN	B: IPA	Time (min)	A: ACN	B: IPA	Time (min)	A: 85% MeOH	B: 97.5% MeOH	Time (min)	A: 85% MeOH	B: 2.5% MeOH
	0	89%	11%	0	89%	11%	0	15%	85%	0	10%	90%
	5.5	62.5%	37.5%	5.5	62.5%	37.5%	9.6	10%	90%	9.6	12.5%	87.5%
	5.51	10%	90%	5.51	10%	90%	19.2	0%	100%	19.2	15%	85%
	6.51	89%	11%	6.51	89%	11%	22	0%	100%	22	15%	85%
	7.5	89%	11%	7.5	89%	11%	25	15%	85%	25	10%	90%

ACN, acetonitrile; IPA, isopropyl alcohol.

**Table 2:** Retention times of five glycidyl ester (GE) species analyzed by different UPLC-ELSD methods.

GE Species	Retention Time (min)			
	Condition 1	Condition 2	Condition 3	Condition 4
C16:0-GE	1.77	2.59	4.9	12.7
C18:0-GE	2.19	3.24	6.9	16.6
C18:1-GE	1.73	2.56	5.1	13.3
C18:2-GE	1.45	2.14	4.0	10.4
C18:3-GE	1.26	1.87	3.3	8.3

**Table 3:** Linearity, coefficients of regression (R^2^), instrumental limit of detection (LOD), and instrumental limit of quantification (LOQ) of five glycidyl ester (GE) species.

GE Species	Equation of Regression Curve	R^2^	Instrumental LOD (µg/mL)	Instrumental LOQ (µg/mL)
C16:0-GE	y = 2.52x^2^ − 102.06x + 939.78	0.9999	2.4	8.0
C18:0-GE	y = 3.43x^2^ − 61.48x + 523.09	1.0000	1.7	5.0
C18:1-GE	y = 4.02x^2^ − 85.29x + 612.33	1.0000	1.7	5.0
C18:2-GE	y = 4.53x^2^ − 114.32x + 912.71	0.9999	1.7	5.0
C18:3-GE	y = 3.41x^2^ − 84.44x + 653.53	0.9999	1.7	5.0

**Table 4:** Inter-day and intra-day precision and accuracy data of five glycidyl ester (GE) species in solvent.

Accuracy	Nominal Concentration (µg/mL)	Analyte				
		C16:0-GE	C18:0-GE	C18:1-GE	C18:2-GE	C18:3-GE
Day 1a	80	99.5	104.4	102.2	101.2	101.7
	50	98.1	104.9	102.1	101.7	101.9
	40	102.5	107.1	104.6	105.3	106.2
Day 1b	80	100.2	104.9	102.3	101.1	101.3
	50	97.2	105.5	102.0	101.5	102.2
	40	102.7	107.3	104.6	105.4	106.0
Intra-day precision	Mean, µg/mL, *n* = 6	100.0	105.7	102.9	102.7	103.2
	CV, %, *n* = 6	2.1	1.1	1.1	1.8	2.0
Day 2	80	103.5	94.3	84.2	102.1	102.2
	50	99.9	91.8	81.3	99.2	98.7
	40	102.1	93.4	82.8	100.7	100.9
Day 3	80	93.4	93.4	97.7	96.8	96.5
	50	91.4	91.4	97.4	96.9	96.6
	40	92.8	92.8	95.6	95.8	95.7
Inter-day precision	Mean, µg/mL, *n* = 12	98.6	99.3	96.4	100.7	100.8
	CV, %, *n* = 12	4.0	6.6	8.6	2.9	3.3

CV, coefficient of variation.

**Table 5:** Intermediate precision and accuracy data of five glycidyl ester (GE) species in oil samples.

Intermediate Accuracy	Nominal Concentration (µg/mL)	Analyte				
		C16:0-GE	C18:0-GE	C18:1-GE	C18:2-GE	C18:3-GE
	40	91.6 ± 11.7	90.9 ± 10.9	88.3 ± 10.3	93.0 ± 5.2	93.6 ± 6.3
Intermediate precision	CV, %, *n* = 5	12.4	12.0	11.6	5.6	6.7
	20	94.2 ± 8.6	107.8 ± 4.3	103.3 ± 1.5	106.8 ± 2.3	101.2 ± 3.1
Intermediate precision	CV, %, *n* = 5	9.1	4.0	1.5	2.1	3.0
^1^ Repeatability	CV, %, *n* = 10	2.1	2.0	2.3	2.6	2.5

CV, coefficient of variation; data are expressed as the mean ± standard deviation (*n* = 5). ^1^ The repeatability of this analysis was obtained by calculating the coefficient of variation (CV) (%) of retention times (*n* = 10).

**Table 6:** Contents of glycidyl esters (GEs) in edible oils determined by the UPLC-ELSD method and Cd 29a-13 GC-MS method.

Oil Sample	GC-MS Method	UPLC-ELSD Method						Relative Percent Difference (%)
	Glycidol Equivalent (µg/g)	C16:0-GE (µg/g)	C18:0-GE (µg/g)	C18:1-GE (µg/g)	C18:2-GE (µg/g)	C18:3-GE (µg/g)	Glycidol Equivalent (µg/g)	
Palm oil (a)	1.63 ± 0.14	5.87 ± 0.33	<LOQ	<LOQ	<LOQ	<LOQ	1.41 ± 0.08	14.47
Palm oil (b)	5.71 ± 0.94	17.42 ± 2.01	<LOQ	3.93 ± 0.68	<LOQ	<LOQ	5.03 ± 0.53	12.66
Rice bran oil	2.96 ± 0.13	<LOQ	<LOQ	7.85 ± 0.11	6.07 ± 0.07	<LOQ	2.64 ± 0.25	11.42
Sunflower oil	0.12 ± 0.02	<LOQ	<LOQ	<LOQ	<LOQ	<LOQ	NA	NA
Safflower oil	0.36 ± 0.02	<LOQ	<LOQ	<LOQ	<LOQ	<LOQ	NA	NA
Canola oil	0.29 ± 0.03	<LOQ	<LOQ	<LOQ	<LOQ	<LOQ	NA	NA
Extra virgin olive oil	<LOQ	<LOQ	<LOQ	<LOQ	<LOQ	<LOQ	NA	NA

LOQ, limit of quantification; NA, not applicable; data are expressed as the mean ± standard deviation (*n* = 3).

## Data Availability

Not applicable.

## Sample Availability

Samples of the glycidyl ester standards and vegetable oils are available from the authors.
